# Stem cell hunt in NHL

**DOI:** 10.18632/oncoscience.234

**Published:** 2015-09-03

**Authors:** Olaf Merkel, Lukas Kenner, Suzanne D. Turner

**Affiliations:** Department of Pathology, University of Cambridge, Addenbrooke's Hospital, Cambridge; European Research Initiative on ALK Related Malignancies (http://www.erialcl.net)

**Keywords:** lymphoma, ALCL, ALK, tumour heterogenity, cancer stem cells

Non-Hodgkin Lymphoma (NHL) is a heterogenous disease consisting of at least 40 different entities that are defined mainly by their clinical and histopathological presentation, their underlying genetic abnormalities but also their presumed cell of origin [1]. The latter can be of B- or T-cell type and derived from different stages of the differentiation process. B-cells are predisposed to DNA alterations including somatic hypermutation, class switch recombination and antigen receptor rearrangement. This innate genetic instability may be one of the reasons why B-cell NHL is the most frequently diagnosed lymphoma in the Western World; many B-cell NHLs arise from germinal center (GC) cells or cells that have just left the GC [2]. Hence, most NHL (including T-cell NHL) are considered to arise from mature, peripheral lymphoid cells. In contrast, both T- and B-lymphoblastic lymphoma/leukaemia cells arise from immature precursors that are found in the thymus and in the bone marrow, respectively. Based on these findings one may hypothesize that the differentiation status of the tumor cell defines their cell of origin in healthy tissue. However, it is also possible that mature lymphoid cells change differentiation status when they become neoplastic and/or that differentiation processes contribute to disease pathogenesis with the seeds of malignancy planted much earlier in their lifespan.

We have shown in a specific entity of T-NHL that some of the tumor cells have a mature cell surface phenotype despite having the ‘ghostly’ genetic appearance of a primitive origin, supporting the idea of cell plasticity in the context of neoplasia [3]. Anaplastic Large Cell Lymphoma (ALCL) is a peripheral T-cell lymphoma; tumour cells are found in the periphery at nodal and extranodal sites, express proteins associated with a cytotoxic T-cell function and exhibit rearrangements of the T-cell receptor (TCR) at the molecular level (the TCR is rarely expressed on the surface of ALCL cells) [3]. However, contrary to popular belief, at least in paediatric ALCL, mediastinal involvement is not infrequent (50%) implicating the thymus, the organ of T-cell development in disease pathogenesis [4]. We show that a gene signature enriched in early thymic progenitors can be detected in a subset of ALCL tumour cells which functionally act as tumour propagating or cancer stem cells (CSC) [3]. The ALCL CSC were isolated using the Side Population (SP) technique which relies on the functional properties of stem cells, i.e. quiescence and expression of efflux pumps presumably evolutionarily conserved to protect the stem cell compartment. This technique has been applied to a number of cancers and was originally developed to enrich for haemopoietic stem cells in murine bone marrow [5]. We show that the ALCL SP cells not only give rise to the bulk tumour population whilst self-replicating to a discrete level but also produce as yet unidentified soluble factors that support the growth of the tumour as a whole [3]. Therefore we hypothesize that a thymic origin may apply to this disease and that these ‘primed’ T-cells egress the thymus and are able to survive and circulate in the periphery to eventually transform as a result of yet to be identified events giving rise to ALCL CSC. Whether distinct clones of ALCL CSC also exist remains to be determined and whilst to date we have been able to identify these cells by means of their functional attributes, in depth mechanistic analyses still need to be performed. It is clear now that at least a limited hierarchy exists and given the postulated thymic origin of these cells it is very likely that tumour propagating cells may reside in the thymus to seed disease relapse, an event that is common in children with ALCL [6].

**Figure 1:**
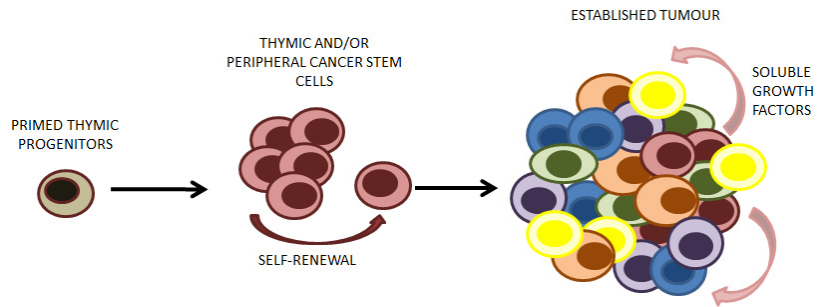
ALCL cancer stem cells (CSC) have a potential thymic origin The CSC not only give rise to the bulk tumour mass that is not able to self-renew like the CSC, but also produce soluble factors that support growth of the whole tumour.
